# Phrenic nerve stimulation, a rare complication of pacemaker: A case report

**DOI:** 10.1097/MD.0000000000025060

**Published:** 2021-03-19

**Authors:** Meddy Dalex, Astrid Malezieux, Thibault Parent, Dina Zekry, Christine Serratrice

**Affiliations:** a Division of Internal Medicine for the Aged; b Division of Geriatrics, Department of Rehabilitation and Geriatrics, Geneva University Hospitals, Geneva, Switzerland.

**Keywords:** complication, older people, pacemaker, phrenic nerve stimulation

## Abstract

**Rationale:**

The phrenic nerve stimulation (PNS) is a rare complication after pacemaker setting. We report a case report that describes this complication and how it can be resolved.

**Patient concerns:**

An 88-year-old man presented himself to the emergency geriatric unit with intermittent painless abdominal contraction due to phrenic nerve stimulation. He has a history of transcatheter aortic valve implantation with cardiac resynchronization therapy pacemaker due to persistent left bundle branch block.

**Diagnoses:**

All the usual causes for abdominal spasms were eliminated and the possibility of a link with the pacemaker was considered. The phrenic nerve stimulation is a rare complication of a pacemaker implantation. It can be clinically nonrelevant but challenging to diagnose for those not familiar with cardiac devices technology.

**Interventions:**

Initial setting was an axis of stimulation between distal left ventricular (LV) and right ventricular. It was changed to LV and D1-M2.

**Outcomes:**

This noninvasive procedure managed to eradicate the involuntary abdominal spasms.

**Lessons:**

PNS could be challenging to diagnose for those not familiar with cardiac devices technology but easy to manage with noninvasive methods.

**KEY POINTS**

The phrenic nerve stimulation is a complication of a pacemaker implantation.

This symptom is generally not very severe, and therefore not well known by general practitioners.

The management of it is usually noninvasive.

## 1 Introduction

Pacemaker implantation is a frequent procedure in older people.^[1]^ Complications can occur in 6% to 12.6% of cases. Common complications include hematomas, pneumothorax, cardiac injury or tamponade, lead dislodgement, deep venous thrombosis, infection and lead or device malfunctions.^[2]^ However rarely, phrenic nerve stimulation (PNS) can occur.

## 2 Case report

The patient, an 88-year-old man, was admitted to the Emergency Geriatrics Unit, in January 2019, after an uncomplicated fall. He lives with his wife in an apartment and was walking with a cane, his frailty score was 4 out of 9 (vulnerable).^[3]^ He was hemodynamically stable.

He has a history of atrial fibrillation, heart failure with normal ejection fraction, hypercholesterolemia, and transcatheter aortic valve implantation with cardiac resynchronization therapy pacemaker (CRT-P) due to persistent left bundle branch block in May 2017 (Quadra Allure MP, St Jude Medical).

At admission, cardiac, lung, neurological examination was unremarkable. However, we noticed visible and palpable intermittent painless abdominal contractions in the left hypochondrium without any evidence of underlying mass or pulses on palpation. The contraction was less intense when he held his breath. The patient had noticed this spasm for a long time, but never bothered him. This spasm was present in decubitus and did not change with position. We did not notice any dyspnea or loss of consciousness.

Blood sample analyses showed no inflammatory syndrome (c-reactive protein < 10 mg/L), normal calcium and magnesium levels (2.33 mmol/L [2.2–2.52] and 0.63 mmol/L [0.59–0.83] respectively). Electrocardiogram was showing an atrial and ventricular pacing. No sign of pulmonary infection or nodal were detected on chest X-ray and pacemaker leads were in place.

After excluding ruptured abdominal aortic aneurysm with a computed tomography, the most likely diagnosis was PNS.

CRT-P, in our patient, was a quadripolar device with 3 leads: 1 in the right atrium (auriculus), 1 in right ventricule (septal), and the last 1 in the left ventricle (lateral coronary sinus vein). Left ventricular (LV) pacing was unipolary mode (2.5 Volts with pulse width of 0.4 msec). Pacing mode was DDD with basal frequency of 70 beats per minute and maximum of 130 beats per minute.

After controlling the pacemaker by a cardiologist, initial setting was an axis of stimulation between distal LV and right ventricular, that was changed to LV and D1-M2 which eradicated the PNS and involuntary spasms disappeared.

Written informed consent for publication of its clinical details was obtained from the patient.

## 3 Discussion

Benign fasciculation syndrome, amyotrophic lateral sclerosis, and metabolic disorders such as hypercalcemia, hypocalcaemia, and hypomagnesemia can cause diaphragmatic contractions.

PNS is frequently reported after CRT-P (around 30%) but clinical impact is relevant in only 3% to 26% of patients.^[4]^ The electrical impulse delivered by the LV lead may incidentally cause PNS.^[5]^ Anatomic explanation of this phenomenon is the left phrenic nerve that passes along the obtuse margin of the left ventricle in 79% of cases and, often, overlapping the left marginal veins.^[6]^ It explains the higher prevalence of PNS with leads implanted in posterolateral veins compared with anterior veins.^[5]^ PNS is usually checked during implantation but cannot always be detected at the moment of implantation, especially that it can only be assessed in the supine position. If LV pacing and PNS threshold are too close, the pacing vector would be changed or the lead moved during implantation.^[7]^ PNS depends on LV lead position, the type of leads, and position of patient. About lead position, Biffi et al ^[8]^ reported more frequent PNS when lead were placed in mid-lateral, mid-posterior, and apical locations versus other LV pacing sites. Juliá et al^[6]^ brought another explanation: they distinguished 2 types of PNS: within the 3 first months after implantation (“early”) and “later” if afterward. Late-PNS appears in 14.8% of patients called super-response (SR) rather than in the non-SR one (2.6%). SR is defined by a decrease of ≥ 30% in LV end-systolic volume at 1 year. Hypothesis of late-PNS apparition in this subgroup is the intense LV reverse remodeling. Body mass index of patients does not influence either PNS or LV pacing thresholds.^[9]^ Management of PNS includes noninvasive and invasive options. The noninvasive option is in reducing the LV pacing output or the LV threshold, increasing the pulse duration up to 1.5 ms, electronic repositioning of bipolar or quadripolar leads. Reducing LV pacing output to 1.5 times LV threshold was determined as the best compromise between eliminating PNS and maintaining an acceptable LV capture safety margin.^[4]^ In patients with quadripolar lead, Behar et al^[10]^ demonstrated a complete elimination of PNS by reprogramming it while bipolar lead required LV lead revision in 40%. Invasive options include surgical reposition of LV lead and isolation of the phrenic nerve by pericardial patch. In 2% to 5% of the cases, the CRT-P needed to be turned off.

In our patient case, PNS appeared approximately 1 year after implantation. Reason for this late appearance was not very clear; minimal lead displacement, and/or cardiac remodeling could be an explanation. It was clinically significant for the patient but easy to manage with the change of pacing vector. Quadripolar devices allow more possibility to eradicate PNS. Early recognition and management is essential.

This clinical case demonstrates the importance of identifying this complication, because of its simplicity of resolution, specifically with noninvasive methods.

## Acknowledgments

The authors thank Anu Mehra and Christopher Inglish for their assistance for English review.

## Author contributions

**Supervision:** Christine Serratrice.

**Validation:** Dina Zekry.

**Writing – original draft:** Meddy Dalex.

**Writing – review & editing:** Astrid Malezieux, Thibault Parent, Dina Zekry, Christine Serratrice.
